# Effects of Ammonium Polyphosphate and Organic Modified Montmorillonite on Flame Retardancy of Polyethylene Glycol/Wood-Flour-Based Phase Change Composites

**DOI:** 10.3390/molecules28083464

**Published:** 2023-04-14

**Authors:** Ke Wang, Chuan Liu, Wenxi Xie, Yihan Ke, Xiaoyong You, Binghao Jing, Yongqian Shi

**Affiliations:** 1 College of Environment and Safety Engineering, Fuzhou University, 2 Xueyuan Road, Fuzhou 350116, China; 2 The Collaborative Innovation Center for Eco-Friendly and Fire-Safety Polymeric Materials (MoE), National Engineering Laboratory of Eco-Friendly Polymeric Materials (Sichuan), State Key Laboratory of Polymer Materials Engineering, College of Chemistry, Sichuan University, Chengdu 610064, China

**Keywords:** ammonium polyphosphate, organic modified montmorillonite, polyethylene glycol, wood flour, phase change materials, flame retardancy

## Abstract

With the depletion of fossil fuel energy and both the slow development and low utilization rate of new eco-friendly energy, finding new ways to efficiently store energy has become a research hotspot. Presently, polyethylene glycol (PEG) is an excellent heat storage material, but it is a typical solid-liquid phase change material (PCM) with a risk of leakage during phase transition. A combination of wood flour (WF) and PEG can effectively eliminate the risk of leakage after the melting of PEG. However, WF and PEG are both flammable materials, which impedes their application. Therefore, it is of great significance to expand their application by forming composites from among PEG, supporting mediums, and flame-retardant additives. This will improve both their flame retardancy and phase change energy storage performance, and will also lead to the preparation of excellent flame-retardant phase change composite materials with solid-solid phase change characteristics. To address this issue, ammonium polyphosphate (APP), organic modified montmorillonite (OMMT), and WF were blended into PEG in specific proportions to prepare a series of PEG/WF-based composites. Both thermal cycling tests and thermogravimetric analysis results demonstrated that the as-prepared composites had good thermal reliability and chemical stability. In addition, during differential scanning calorimetry tests, the PEG/WF/8.0APP@2.0OMMT composite presented the highest melting latent heat (176.6 J/g), and its enthalpy efficiency reached more than 98.3%. The PEG/WF/8.0APP@2.0OMMT composite also exhibited superior thermal insulation performance when compared to the pure PEG/WF composite. Furthermore, the PEG/WF/8.0APP@2.0OMMT composite exhibited a significant 50% reduction in peak heat release rate as a result of the synergistic effect between OMMT and APP in the gas and condensed phases. This work offers a useful strategy for the fabrication of multifunctional phase-change material, which is expected to broaden its industrial applications.

## 1. Introduction

Polyethylene glycol (PEG) is well known for its nontoxic, biocompatible, and biodegradable properties, as well as for its favorable chemical and thermal stability and adjustable average molecular weight [1,2,3,4,5]. However, PEG has a risk of leakage during phase transition because of solid-liquid phase transition [6,7], limiting its wide application. Therefore, improving the fire safety of PEG will significantly broaden its application in many fields.

Wood flour (WF), with high porosity and cavity, has been widely applied in the market of filled polymers [8,9,10]. The advantages of WF include its renewable nature, low density, high stiffness, abundant supply, and low cost [11,12]. In terms of microstructure, wood material contains many tubular channels along the wood growth and is made of a natural structure of cellulose fibers with rich hydroxyl groups, which provides and enhances the polymer absorption capability of WF. Jiang et al. incorporated WF into PEG with an encapsulation ratio of 52.8 wt.% and found that the synthesized PEG/WF composites provided superior thermal stability during 200 cycles of melting and freezing, and that it did so without leaking [13,14]. In addition, in the timber industry more than half of each log, including scrap wood and sawdust, is wasted [15]. Therefore, the combination of WF and PEG may not only effectively solve the problem of PEG leaking out after melting, but it may also improve the utilization of wood residues.

However, it has been reported that both WF and PEG are flammable; as a result, excellent flame-retardant phase change composites need to be developed [16,17]. Ammonium polyphosphate (APP), an intumescent flame retardant, has shown wide application in many polymer-based materials due to both its low toxicity and low cost [18,19,20]. In addition, APP has been used as a filler to modify wood powder. For example, Wang et al. blended either APP or microencapsulated APP (MCAPP) with WF and polypropylene (PP) to manufacture WF/PP composites (WPCs) via hot pressing. Their results indicated that APP was successfully coated by cross-linked beta-cyclodextrin [21]. It was found that the synergistic charring effect between APP and WF facilitated the formation of a protective char layer during the polymer combustion, which reduced heat and oxygen diffusion [22]. However, APP has the disadvantage of weak interfacial adhesion with the polymer matrix. Therefore, it is necessary to modify APP to enhance its interfacial interaction with WF composites.

In recent years, montmorillonite (MMT) has become one of the most widely used clay minerals due to both its natural abundance and beneficial properties (high cation exchange capacity, high specific surface area, and large aspect ratio [23,24]). It is a type of natural clay mineral that has a layered structure, consisting of two silica tetrahedral sheets sandwiching an edge-shared octahedral sheet of either aluminum or magnesium hydroxide [25,26]. The introduction of MMT into a mixture of WF and polymers can improve its mechanical properties [27]. Compared to natural MMT, organic modified montmorillonite (OMMT) may endow polymer composites with better mechanical and physical properties due to its superior dispersion [28]. Typically, OMMT is produced by ion-exchange reactions between the naturally occurring cations in MMT and cationic surfactants such as alkyl ammonium. This surface modification promotes both new interface characteristics and large inter-gallery distances [29,30]. Consequently, OMMT serves as a better filler than natural MMT in achieving high-performance composites. For example, Wang et al. added OMMT into either WF or WF/polylactic acid composites and subsequently observed improvements in mechanical properties [31]. Many researchers have reported that the addition of OMMT decreased the peak heat release rate (PHRR) of composites [32,33,34]. It has also been demonstrated that OMMT and APP have a synergistic flame-retardant effect [35].

Given the synergistic flame-retardant effect between OMMT and APP, it is likely that the flame-retardant properties of PCM will be improved with the loading of APP and OMMT. In this work, PEG/WF-based flame-retardant phase change composites were synthesized via vacuum adsorption and hot pressing, and thermal stability, flammability, and other composite properties were analyzed. In addition, the mechanisms for reinforcing the flame retardancy of PCM composites were discussed.

## 2. Results and Discussion

### 2.1. Morphology and Structure Characterization of PEG/WF and Its Composites

**Figure 1:**
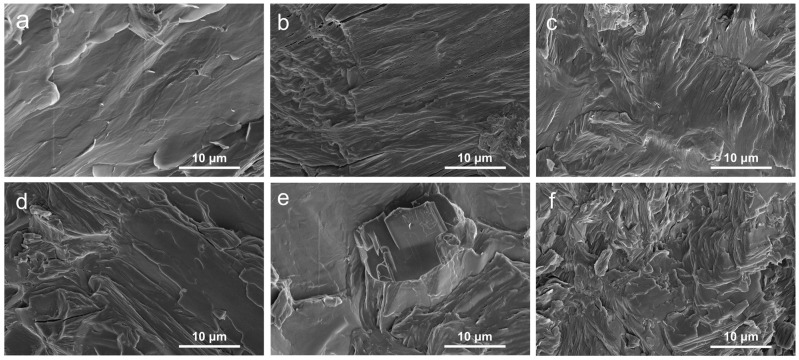
SEM images of the fracture surface of (**a**) PEG/WF, (**b**) PEG/WF/6.0APP@4.0OMMT, (**c**) PEG/WF/8.0APP@2.0OMMT, (**d**) PEG/WF/9.0APP@1.0OMMT, (**e**) PEG/WF/10.0APP, and (**f**) PEG/WF/10.0OMMT composites.

An SEM instrument was used to examine the fracture morphology of the samples, as exhibited in Figure 1 The surface of pure PEG/WF was relatively smooth with typically brittle fractures (See Figure 1a), implying efficient absorption of PEG into WF. Additionally, the chemical structure of WF includes rich hydroxyl groups in cellulose molecular chains [14], which result in the strong intermolecular hydrogen bonding interactions between PEG and WF. Therefore, PEG can be well impregnated into WF and avoid WF leakage [13]. After the incorporation of APP, the surface of PEG/WF/10.0APP began to roughen, and agglomeration was observed (See Figure 1e). This result can be ascribed to the poor dispersion of APP in the PEG/WF composite. As shown in Figure 1b–d, many protrusions were embedded into the PEG/WF composite with the addition of APP and OMMT, indicating the strong interface adhesion among APP, OMMT, and PEG/WF composite; the dispersion of APP and OMMT in the PEG/WF composite was also improved. Figure 1b–d,f show the SEM images of the PEG/WF composites with 10.0 wt.%, 4.0 wt.%, 2.0 wt.%, and 1.0 wt.% OMMT: the bright regions are soft segments and the darker regions are hard segments. After the addition of OMMT, the surface of the PEG/WF composites became rough and had obvious cracks. This is likely because the soft segment was limited by the interaction of the interface between the matrix and the microphase separation, which led to ineffective covering of the surface of the composites. OMMT was uniformly dispersed in the PEG/WF matrix and had no obvious inorganic particles on the fracture surface, which may indicate an exfoliated OMMT structure [36,37].

### 2.2. Flame Retardancy Evaluation of PEG/WF and Its Composites

**Figure 2:**
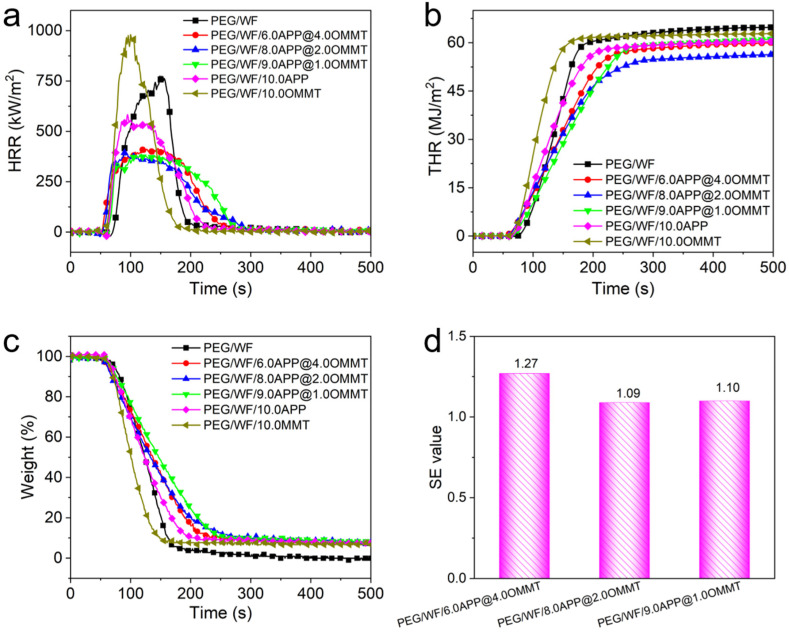
(**a**) HRR, (**b**) THR, (**c**) weight, and (**d**) SE values of PEG/WF and its composites obtained from cone calorimetry.

Cone calorimetry has been widely employed to investigate the combustion behavior of polymers under specific real-world fire conditions [38,39,40,41]. In this study, the combustion properties of PGF/WF and its composites were measured; the combustion results are shown in Figure 2 and Table 1. As can be seen in Table 1, the time to ignition (TTI) of all PEG/WF composites was lower than that of pure PEG/WF, which can be ascribed to the degradation of PEG/WF catalyzed by flame retardants. This result was further confirmed by the TGA results in Figure 2a. The PHRR is considered one of the most important parameters to evaluate the fire safety properties of polymers. As shown in Figure 2a, the PHRR of pure PEG/WF was as high as 795 kW/m^2^, indicating its intrinsically high flammability. Compared to that of pure PEG/WF, the PHRR value decreased by 25.8% with the addition of 10 wt.% APP, while the PHRR value of PEG/WF increased by 24.4% with the addition of 10 wt.% OMMT. Notably, when both APP and OMMT were added and the ratio between APP and OMMT increased, the PHRR values of the PEG/WF composites decreased further. For example, the PHRR values of PEG/WF/9.0APP@1.0OMMT and PEG/WF/8.0APP@2.0OMMT were both significantly reduced by 50%, as compared to that of pure PEG/WF (See Table 1). That the addition of OMMT increased the PHRR mainly results from the higher OMMT content. Specifically, the layered structure of OMMT hinders the escape of water vapor and other gases produced by the flame-retardant system, which is not conducive to the expansion and foaming of the melting system. Moreover, the intercalator of OMMT decomposes early, and the released chloride ion indues the decomposition of the flame retardant in advance, thus reducing the efficiency of the flame retardant [37]. 

The total heat release (THR) had a similar trend to that of the PHRR, and PEG/WF/8.0APP@2.0OMMT exhibited the lowest THR value (56.3 MJ/m^2^), which was 13% lower than that of the control sample (See Figure 2b). One more parameter that is crucial to assessing the flammability of structures is the maximum average release heat emission (MARHE), which is considered a good measure of the propensity for fire development in a real-world fire situation [42,43]. Using HRR, both the average rate of heat emission (ARHE) defined in Equation (1) [44] and its maximum (MARHE) were calculated.

(1) $$\left(t_{n}\right)=\frac{\sum_{2}^{n}\left(t_{n}-t_{n-1}\right)\times \frac{\left(q_{n}-q_{n-1}\right)}{2}}{\left(t_{n}-t_{0}\right)}$$

where $t_{0}$ is the time at the beginning of the test, $t_{n}$ is test time after $t_{0}$, and $q_{n}$ is the rate of heat release at $t_{n}$. The cone calorimeter machine showed MARHE values of 313.2 kW/m^2^ and 289.9 kW/m^2^ for the pure PEG/WF and PEG/WF/10.0APP, respectively, which implies that APP additives can decrease the MARHE of the composite by 7.4%. Furthermore, MARHE values of PEG/WF containing APP/OMMT blends were reduced by ca. 19.3%, 25.9% and 25.6%, respectively, compared with that of pure PEG/WF, showing the greater flame-retardant efficiency of APP/OMMT. The lowest MARHE value was obtained with the addition of 8.0 wt.% APP and 2.0 wt.% OMMT by 25.9%, which is also consistent with the THR results. The char residues of PEG/WF and its composites are shown in Figure 2c. Only a small amount of char residue (0 wt.%) was left by pure PEG/WF. However, the char residues of the PEG/WF composites were strikingly improved. Specifically, the PEG/WF/8.0APP@2.0OMMT showed the highest char residue (7.9 wt.%) among all the PEG/WF composites. These results further confirmed the excellent synergistic effect between APP and OMMT in improving the fire resistance of PEG/WF composites.

To quantify the synergistic effect (SE) between APP and OMMT, the SE values were calculated based on Equation (2) [45]; the results are illustrated in Figure 2d.

(2) $$\mathrm{SE}=\frac{1-\Delta M\left(PEG/WF/xAPP@yOMMT\right)}{1-\left(1-\frac{x}{\left(x+y\right)}\left(1-\Delta M\left(PEG/WF/\left(x+y\right)APP\right)\right)\right)\left(1-\frac{x}{\left(x+y\right)}\left(1-\Delta M\left(PEG/WF/\left(x+y\right)OMMT\right)\right)\right)}$$

where *x* and *y* represent the weight fractions of APP and OMMT, respectively, in PEG/WF composites. ΔM is the improvement in a specific fire property such as THR.

As can be seen in Figure 2d, the SE values of THR for PEG/WF/6.0APP@4.0OMMT, PEG/WF/8.0APP@2.0OMMT, and PEG/WF/9.0APP@1.0OMMT were 1.27, 1.09, and 1.10, respectively. This result indicates the superior synergistic effect between APP and OMMT in improving the flame retardancy of PEG/WF composites.

**Figure 3:**
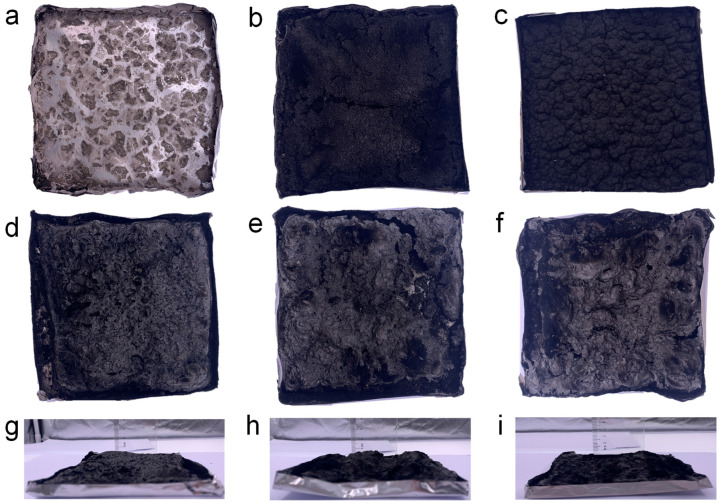
Digital photographs of (**a**) PEG/WF, (**b**) PEG/WF/10.0APP, (**c**) PEG/WF/10.0OMMT, (**d**,**g**) PEG/WF/6.0APP@4.0OMMT, (**e**,**h**) PEG/WF/8.0APP@2.0OMMT and (**f**,**i**) PEG/WF/9.0APP@1.0OMMT composites after the cone test.

To examine the flame-retardant mechanism of the composites, both the morphology and structure of the residual carbon were analyzed. Digital photographs of the char residues of PEG/WF and its composites are shown in Figure 3 It is clear from Figure 3a that scarcely any char residue remained after the combustion of PEG/WF. When either APP or OMMT was introduced, the char yield of the PEG/WF composites increased; however, no intumescent characteristics can be observed in the char layer of PEG/WF/10.0APP or PEG/WF/10.0MMT (See Figure 3b,c). In contrast, when both APP and OMMT were added to PEG/WF/6.0APP@4.0OMMT, PEG/WF/8.0APP@2.0OMMT, and PEG/WF/9.0APP@1.0OMMT, these composites displayed an obvious increase in char residue; the char residue became more compact as APP increased (See Figure 3d–f). The distinctly intumescent residual carbon of PEG/WF/6.0APP@4.0OMMT, PEG/WF/8.0APP@2.0OMMT, and PEG/WF/9.0APP@1.0OMMT can be observed from the side view of the residual carbon (See Figure 3g–i). This phenomenon indicates the synergistic effect of APP and OMMT in improving carbon quality. These char layers act as a physical barrier to prevent heat transfer, smoke generation, and toxic gas release during combustion, and thus the fire safety of PEG/WF is remarkably improved.

**Figure 4:**
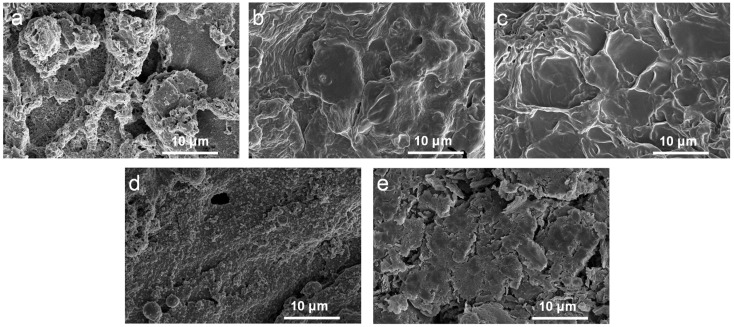
SEM images of the char residues of (**a**) PEG/WF/6.0APP@4.0OMMT, (**b**) PEG/WF/8.0APP@2.0OMMT, (**c**) PEG/WF/9.0APP@1.0OMMT, (**d**) PEG/WF/10.0APP and (**e**) PEG/WF/10.0OMMT composites after cone test.

The morphology of the char layer was investigated using SEM, as shown in Figure 4. The samples showed a relatively continuous, dense carbon layer with the addition of APP@OMMT hybrids. However, the PEG/WF/6.0APP@4.0OMMT sample displayed many holes and cracks. In contrast, the char layers of PEG/WF/8.0APP@2.0OMMT and PEG/WF/9.0APP@1.0OMMT had a continuous and compact structure. Those residual chars act as a physical barrier to protect the underlying PEG/WF substrate from burning and inhibit the transfer of heat [41,46,47], thus enhancing the flame retardancy of PEG/WF composites. These results support those previously seen in the digital photos (See Figure 3).

### 2.3. Phase Change Performance of PEG/WF and Its Composites

**Figure 5:**
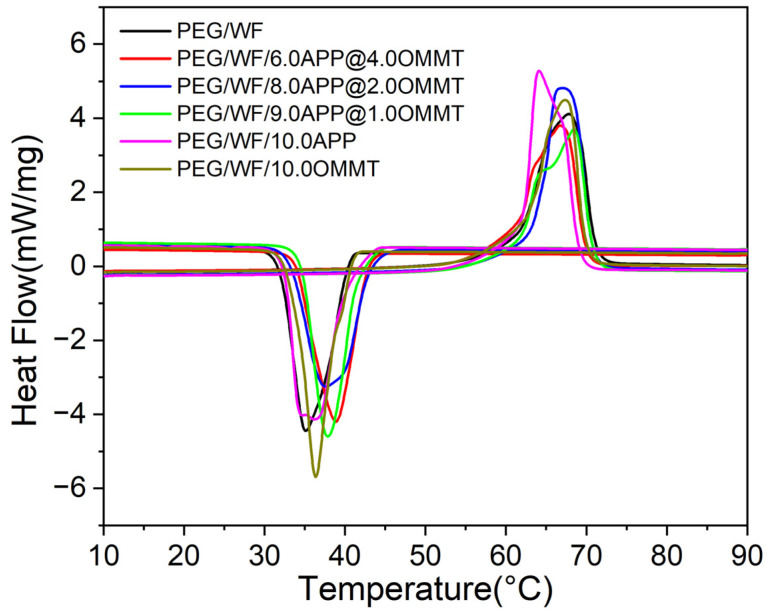
DSC curves of PEG/WF and its composites.

The DSC is used to characterize the phase change temperature and phase change energy of materials [48]. The DSC curves of the PEG/WF phase change composites are shown in Figure 5. The corresponding detailed melting point ($T_{m}$), freezing point ($T_{f}$), melting latent heat ($\Delta H_{m}$), and freezing latent heat ($\Delta H_{f}$) are summarized in Table 2. As shown in Figure 5, all samples had the same melting and freezing process, and the phase transition temperature ranged from 10 °C to 90 °C. However, with the addition of different proportions of flame retardants, the amplitude of the melting and freezing curves of the composites changed significantly compared with those of the pure PEG/WF samples. During the melting process, the melting peak values of PEG/WF/10.0APP and PEG/WF/8.0APP@2.0OMMT were clearly higher than that of pure PEG/WF. The latent heat of the phase change of composites is obtained via the area of the endothermic peak and the exothermic peak, as presented in Table 2. With the addition of APP and OMMT, the fusion latent heat values of PEG/WF/6.0APP@4.0OMMT, PEG/WF/8.0APP@2.0OMMT and PEG/WF/9.0APP@1.0OMMT were lower than that of pure PEG/WF. In addition, the $T_{m}$ values of all the samples (except for PEG/WF/6.0APP@4.0OMMT) were higher than that of pure PEG/WF. In a strong interaction in PEG/WF phase change materials, $T_{m}$ increases, whereas $T_{m}$ decreases with a weak interaction [49,50]. Therefore, the $T_{m}$ value of PEG/WF/8.0APP@2.0OMMT was the highest among all the samples, showing the strongest interaction.

To further investigate the change of the latent heat of PEG/WF composites, the relative latent heat efficiency (η) was calculated by using Equation (3) [51]; the results are listed in Table 3.

(3) η=ΔHm,compositesΔHm,PEG/WF×100%

where $\Delta H_{m,composites}$ and $\Delta H_{m,PEG/WF}$ represent the melting latent heat value of prepared composites and pure PEG/WF samples, respectively. A larger η value indicates a smaller latent heat loss [48]. The PEG/WF/10.0APP composite displayed the largest η (102.9%) among all of specimens. In addition, PEG/WF/8.0APP@2.0OMMT showed a higher η value than the other APP/OMMT composites. These results indicate that adding appropriate proportions of APP and OMMT can effectively reduce the latent heat loss of PEG/WF.

**Figure 6:**
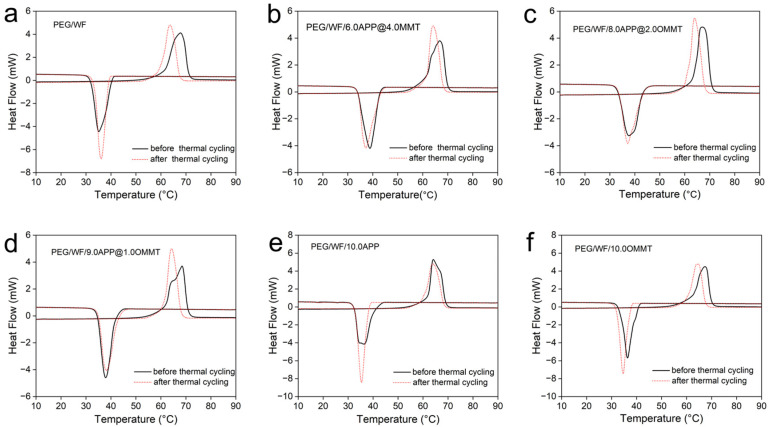
DSC curves of (**a**) PEG/WF, (**b**) PEG/WF/6.0APP@4.0OMMT, (**c**) PEG/WF/8.0APP@2.0OMMT, (**d**) PEG/WF/9.0APP@1.0OMMT, (**e**) PEG/WF/10.0APP and (**f**) PEG/WF/10.0OMMT composites before and after thermal cycling.

The DSC curves before and after thermal cycling of the prepared composites are shown in Figure 6a–f, and the related data are recorded in Table 4. Compared to that of the pure PEG/WF sample, both the phase transition temperatures and latent heat of PEG/WF/6.0APP@4.0OMMT, PEG/WF/8.0APP@2.0OMMT and PEG/WF/9.0APP@1.0OMMT composites changed slightly after the thermal cycle. These results demonstrate that the as-prepared PEG/WF/APP@OMMT samples have good thermal reliability in terms of phase transition properties after a thermal cycle.

### 2.4. Thermal Stability Analysis of PEG/WF and Its Composites

**Figure 7:**
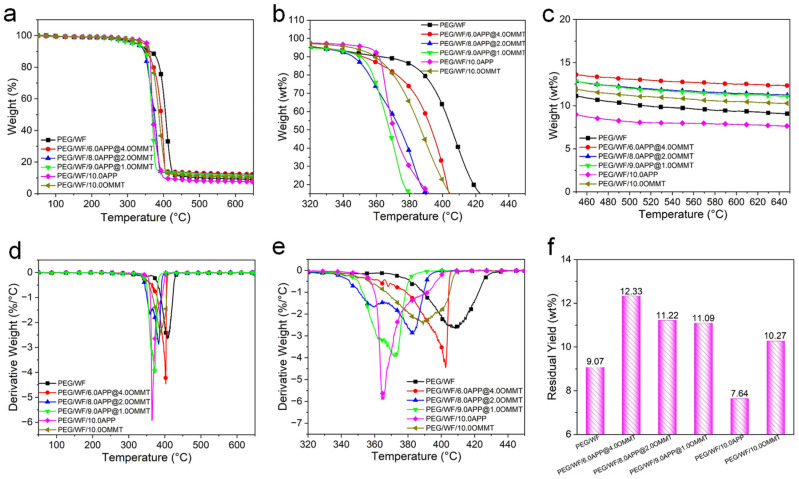
TG (**a**–**c**), DTG (**d**,**e**) curves and char yield (**f**) at 650 °C of PEG/WF and its composites.

Thermal stability is a significant factor of PCMs due to the limiting usability of thermal degradation. The TGA technique is usually employed to evaluate the thermal stability of polymers [52,53]. The TG and derivative TG (DTG) curves of the composites are shown in Figure 7, and the related data are summarized in Table 5. As is evident in the DTG curves, only one-stage decomposition occurred in pristine PEG/WF, PEG/WF/10.0APP, and PEG/WF/10.0OMMT. However, with the incorporation of the APP@OMMT hybrid, the PEG/WF composites underwent a two-stage thermal degradation process. The first stage (which ranged from 340 °C to 365 °C) can be attributed to the decomposition of APP, while the second stage (between 370 °C and 440 °C) occurred due to the degradation of OMMT and PEG/WF. All samples showed little weight loss when the temperature was lower than 200 °C, whereas significant weight loss occurred when the temperature exceeded 500 °C.

The pure PEG/WF began to degrade at 325 °C and decomposed rapidly at 408 °C. After the addition of APP and OMMT, the thermal decomposition process of the composites was the same as that of pure PEG/WF. Compared to pure PEG/WF, PEG/WF/6.0APP@4.0OMMT, PEG/WF/8.0APP@2.0OMMT and PEG/WF/9.0APP@1.0OMMT composites had lower values of T_5%_, T_50%_ and T_max_, mainly resulting from the decomposition of APP and OMMT at lower temperatures. However, the residual yield data of PEG/WF/6.0APP@4.0OMMT, PEG/WF/8.0APP@2.0OMMT and PEG/WF/9.0APP@1.0OMMT composites increased compared with that of pure PEG/WF. Moreover, with the change of the proportion of APP@OMMT hybrids, the residual yield of the composites also changed regularly, indicating that APP and OMMT play a synergistic role in improving the quality of char residues during the decomposition of the composites. Additionally, the PEG/WF/10.0APP reached both the lowest maximum weight loss rate and the lowest residual yield at 650 °C among all the PEG/WF composites, which implies that APP accelerates the degradation of PEG/WF.

These results indicate that the as-prepared composites display excellent thermal stability in the temperature range of phase transition, which is beneficial for thermal energy storage applications.

### 2.5. Temperature Regulation Performance of PEG/WF and Its Composites

**Figure 8:**
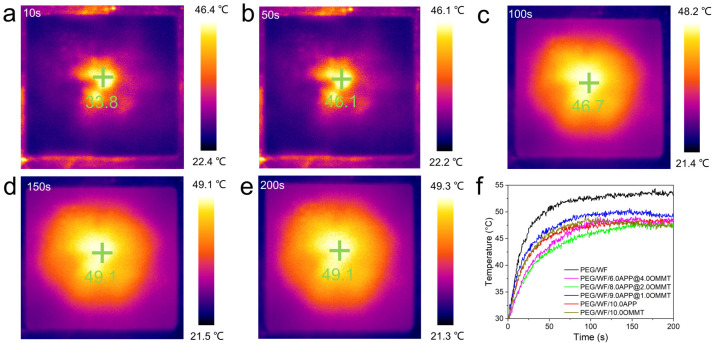
Representative thermal images of photothermal performance (**a**–**e**) and temperature-time curves of samples (**f**).

As shown in Figure 8a–e, the temperature regulation performance of the samples was observed and recorded by an infrared thermal imaging camera. The yellow and black portions represent high and low temperatures, respectively. At any given moment, the temperature near the center of the material was the highest. At the beginning of heating (10 s), the image was generally black. As the heating temperature continued to rise (50 s), the temperature of the sample increased. When the heating time exceeded 100 s, the image was mostly yellow. Finally, the color of the image gradually turned yellow during the heating process.

**Figure 9:**
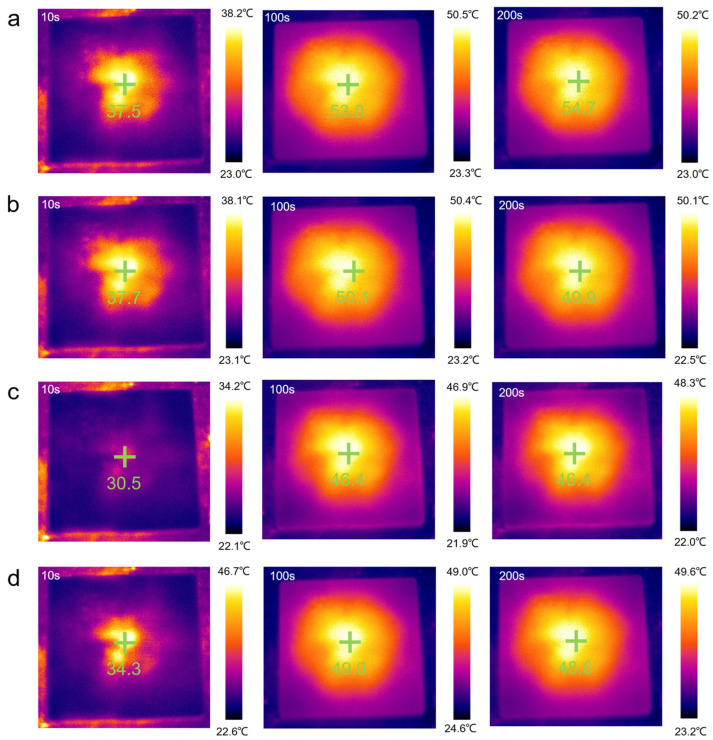
Thermal imaging camera results of the (**a**) PEG/WF, (**b**) PEG/WF/9.0APP@1.0OMMT, (**c**) PEG/WF/8.0APP@2.0OMMT and (**d**) PEG/WF/6.0APP@4.0OMMT composites.

The results shown in Figure 8f are consistent with the temperature-time curve result. Over time, the temperature of all the samples increased, but compared to the rate of temperature increase of pure PEG/WF, the rates of all the composites were relatively slow. In addition, the final heating temperatures of all composites were reduced, which can also be seen in Figure 9. Under the same conditions, the temperature of the samples containing APP and OMMT rose more slowly. Therefore, in addition to the pure PEG/WF sample, the other PEG/WF composites showed good thermal insulation performance. Notably, both the rate of temperature increase and the final heating temperature of the PEG/WF/8.0APP@2.0OMMT material were significantly reduced, which reflects its excellent thermal insulation performance, and suggests that it can be used as a kind of thermal insulation material.

**Figure 10:**
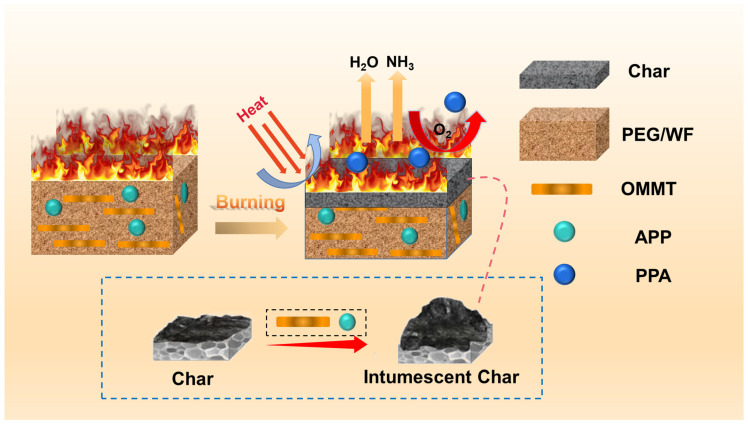
Schematic illustration of the flame retardant mechanism of RPUF composites.

Based on the above discussion, a flame-retardant mechanism is proposed, as illustrated in Figure 10. As PEG/WF decomposes, the incorporation of APP induces the carbonization of cellulose in WF. During the reaction between APP and cellulose, cellulose acts as a carbon source while APP produces phosphoric acid (PPA), which serves as a foaming agent that encourages intumescent char formation [54]. In addition, the surface ions on the OMMT layers may complex with the oxygen in P=O and P-O groups, weakening the bonding of -O-${\mathrm{NH}}_{4}^{+}$ and -OH and allowing APP to release NH_3_ and H_2_O more easily. The synergistic charring effect between APP and OMMT accelerates the formation of a polyphosphoric acid cross-linking network and thereby inhibits the degradation of PPA, which then increases the amount of residue in the condensed phase [55,56,57]. When subjected to thermal expansion, the surface of the substrate is covered by carbonaceous char with a strengthened structure, and the air and heat are isolated to improve the flame retardancy of WF/PEG. At the same time, the released non-flammable gases (such as NH_3_ and H_2_O) play a role in diluting the concentration of oxygen and retarding the spreading of the heating process [58,59]. Therefore, the multiple effects of APP and OMMT significantly improve the fire safety of PEG/WF composites via both the gas and condensed phase mechanisms.

## 3. Materials and Methods 

### 3.1. Materials

Polyethylene glycol (PEG, laboratory reagent (LR), M_n_ = 10,000), a white waxy crystalline solid powder, was purchased from Shanghai Aladdin Bio-Chem Technology Co., Ltd. (Shanghai, China). Rosewood flour (WF, 200 mesh) was obtained from the Xianyou Hongjingtian Mahogany furniture factory (Fujian, China). Ammonium polyphosphate (APP) was provided by Shandong Chengxu New Material Co., Ltd (Shandong, China). Organic modified montmorillonite (OMMT, DK2) was supplied by Zhejiang Fenghong New Materials Co., Ltd. (Zhejiang, China).

### 3.2. Preparation of PEG/WF and Its Composites

**Figure 11:**
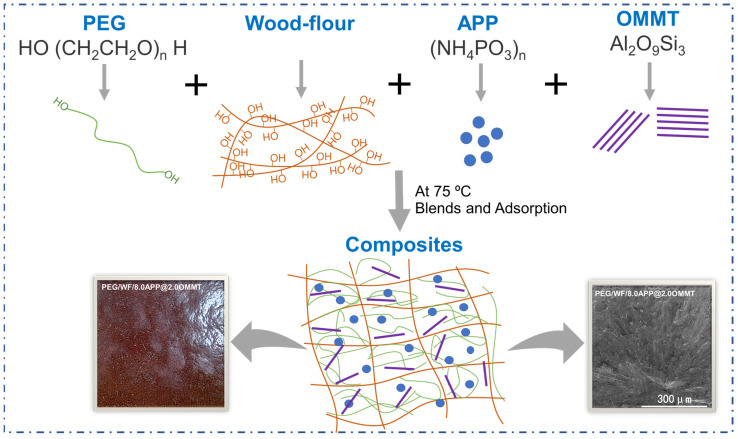
The synthetic route of PEG/WF composites.

Firstly, PEG was melted at 75 °C. Next, WF and APP/OMMT were added to the melted PEG through vigorous stirring at 75 °C. After full blending, the filtration was carried out for 30 min. Then the samples were obtained using a small vulcanizing machine at 75 °C and 10 MPa. To investigate the effect of APP/OMMT hybrids on the performance of PEG/WF-based phase change composites, the APP/OMMT content was adjusted by changing the mass ratio. In summary, a total of six groups of samples were involved, including a control group (1^#^) and five experimental groups (2^#^, 3^#^, 4^#^, 5^#^, 6^#^); the specific formulas are shown in Table 6, and the synthetic route of the material is shown in Figure 11. For comparison, PEG/WF composites without OMMT or APP were prepared accordingly.

### 3.3. Instruments and Measurements

The morphologies and microstructures of materials were investigated using scanning electron microscopy (SEM, FEI Nova Nano SEM 230, FEI CZECH REPUBLIC S.R.O., Brno, Czech Republic). All samples were gold sputtered to prevent the occurrence of electrostatic charge during observation.

Both the light-thermal conversion performance and the temperature-regulated performance of the prepared materials were evaluated via simulated solar irradiation using a MAG-F6-type infrared thermography camera (MAGNITY TECHNOLOGIES Co., Ltd., Shanghai, China).

The thermal storage performance of the prepared materials was measured by a differential scanning calorimeter (DSC, Netzsch, Germany) under a nitrogen atmosphere at a heating and cooling rate of 10 °C/min. Before the testing process, the samples were heated from 0 to 100 °C and kept at 100 °C for 5 min to eliminate any thermal history.

Thermogravimetric analysis (TGA) was carried out with a Q5000 thermal analyzer (TA Co., New Castle, DE, USA) under a flow rate of 100 mL/min. The measurements were performed in a temperature range of 50–750 °C with a heating rate of 20 °C/min under a nitrogen atmosphere.

The flame retardancy of the samples was evaluated using a TTech-GBT16172-2 type cone calorimeter (TESTech, Suzhou, China) under an external heat flux of 35 kW/m^2^. The dimensions of each sample were 100 × 100 ×3 mm^3^. Each sample was tested three times to obtain an average value. All of the samples were wrapped with a layer of aluminum foil.

## 4. Conclusions

In this work, flame-retardant PEG/WF-based phase change composites were synthesized via vacuum adsorption and hot pressing. The obtained results demonstrated that PEG/WF composites possessed remarkable flame retardancy and thermal stability. The TGA results indicated that the thermal stability of PEG/WF composites was significantly improved after combining PEG/WF with APP@OMMT hybrids. The cone calorimeter test showed that the PEG/WF/9.0APP@1.0OMMT and PEG/WF/8.0APP@2.0OMMT composites had a significant reduction in PHRR (50%) and the lowest THR value (56.3 MJ/m^2^), which was 13% lower than that of pure PEG/WF. Furthermore, the digital photographs of char residues demonstrated that when APP and OMMT were added, the distinctly intumescent residual carbon of PEG/WF/6.0APP@4.0OMMT, PEG/WF/8.0APP@2.0OMMT, and PEG/WF/9.0APP@1.0OMMT was observed. In this manner, the fire safety of PEG/WF was remarkably improved. Specifically, the PEG/WF/8.0APP@2.0OMMT composite showed the highest melting latent heat (176.6 J/g) among all the samples, and its enthalpy efficiency reached more than 98.3%; this material also exhibited excellent thermal insulation performance. This improvement in fire safety was attributed to the multiple flame-retardant mechanisms of APP@OMMT in the condensed and gas phases. This work suggests an innovative strategy for the design of multifunctional PCM, which is expected to broaden its industrial application.

## Figures and Tables

**Table 1:** Cone calorimetric data for PEG/WF and its composites (heat flux of 35 kW/m^2^).

Sample No.	TTI (s)	PHRR (kW/m^2^)	THR (MJ/m^2^)	MARHE (kW/m^2^)	Char Residues (wt.%)
Error	±3.6	±24	±1.1	±27.4	±1.8
PEG/WF	66	795	64.8	313.2	0.0
PEG/WF/6.0APP@4.0OMMT	50	425	60.0	252.6	7.6
PEG/WF/8.0APP@2.0OMMT	51	399	56.3	232.1	7.9
PEG/WF/9.0APP@1.0OMMT	59	397	61.2	233.0	7.5
PEG/WF/10.0APP	58	590	60.4	289.9	7.4
PEG/WF/10.0OMMT	53	989	62.8	388.9	6.7

**Table 2:** Latent heat storage properties of PEG/WF and its composites.

Sample No.	Melting Process		Freezing Process	
	$T_{m}$ (°C)	$\Delta H_{m}$ (J/g)	$T_{f}$ (°C)	$\Delta H_{f}$ (J/g)
PEG/WF	61.3	179.7	40.5	153.1
PEG/WF/6.0APP@4.0OMMT	61.2	163.9	42.9	147.4
PEG/WF/8.0APP@2.0OMMT	63.7	176.6	43.2	155.7
PEG/WF/9.0APP@1.0OMMT	61.9	159.7	41.8	145.4
PEG/WF/10.0APP	62.1	185.0	40.2	168.6
PEG/WF/10.0OMMT	62.6	166.7	39.7	148.3

**Table 3:** Thermal energy storage characteristics of different composites in the present study.

Sample No.	$\Delta H_{m,\;composites}$ (J/g)	$\Delta H_{m,PEG/WF}$ (J/g)	*η* (%)
PEG/WF/6.0APP@4.0OMMT	163.9	179.7	91.2
PEG/WF/8.0APP@2.0OMMT	176.6	179.7	98.3
PEG/WF/9.0APP@1.0OMMT	159.7	179.7	88.9
PEG/WF/10.0APP	185.0	179.7	102.9
PEG/WF/10.0OMMT	166.7	179.7	92.8

**Table 4:** Latent heat storage properties of PEG/WF and its composites after thermal cycling.

Sample No.	Melting Process		Freezing Process	
	$T_{m}$ (°C)	$\Delta H_{m}$ (J/g)	$T_{f}$ (°C)	$\Delta H_{f}$ (J/g)
PEG/WF	60.0	161.9	39.0	153.6
PEG/WF/6.0APP@4.0OMMT	61.5	151.8	43.2	146.8
PEG/WF/8.0APP@2.0OMMT	61.3	161.6	43.8	155.2
PEG/WF/9.0APP@1.0OMMT	61.5	150.2	42.8	145.9
PEG/WF/10.0APP	60.5	174.4	38.0	169.1
PEG/WF/10.0OMMT	60.3	154.3	37.1	146.7

**Table 5:** TGA data of PEG/WF and its composites.

Sample No.	T_5%_ (°C)	T_50%_ (°C)	T_max_ (°C)	Residual Yield at 650 °C (wt.%)
PEG/WF	325	405	408	9.07
PEG/WF/6.0APP@4.0OMMT	324	393	402	12.33
PEG/WF/8.0APP@2.0OMMT	320	374	383	11.22
PEG/WF/9.0APP@1.0OMMT	318	367	371	11.09
PEG/WF/10.0APP	351	370	364	7.64
PEG/WF/10.0OMMT	343	386	390	10.27

Notes: T_5%_ represents the temperature at 5% weight loss; T_50%_ represents the temperature at 50% weight loss; T_max_ denotes the temperature at the maximum weight loss rate.

**Table 6:** Specific formulation of PEG/WF hybrid phase change composites (wt.%).

No.	Sample No.	PEG	WF	APP	OMMT
1^#^	PEG/WF	83.3	6.7	0.0	0.0
2^#^	PEG/WF/6.0APP@4.0OMMT	83.3	6.7	6.0	4.0
3^#^	PEG/WF/8.0APP@2.0OMMT	83.3	6.7	8.0	2.0
4^#^	PEG/WF/9.0APP@1.0OMMT	83.3	6.7	9.0	1.0
5^#^	PEG/WF/10.0APP	83.3	6.7	10.0	0.0
6^#^	PEG/WF/10.0OMMT	83.3	6.7	0.0	10.0

## Data Availability

The data presented in this study are available on request from the corresponding author. The data are not publicly available due to privacy.

## Sample Availability

Samples of the PEG/WF, PEG/WF/10.0APP, PEG/WF/10.0OMMT, PEG/WF/6.0APP@4.0OMMT, PEG/WF/8.0APP@2.0OMMT and PEG/WF/9.0APP@1.0OMMT are available from the authors.

## References

[1] Liu Z., Zhang Y., Hu K., Xiao Y., Wang J., Zhou C., Lei J. (2016). Preparation and properties of polyethylene glycol based semi-interpenetrating polymer network as novel form-stable phase change materials for thermal energy storage. Energy Build..

[2] Lu X., Fang C., Sheng X., Zhang L., Qu J. (2019). One-Step and Solvent-Free Synthesis of Polyethylene Glycol-Based Polyurethane As Solid–Solid Phase Change Materials for Solar Thermal Energy Storage. Ind. Eng. Chem. Res..

[3] Lu X., Huang J., Kang B., Yuan T., Qu J.-p. (2019). Bio-based poly (lactic acid)/high-density polyethylene blends as shape-stabilized phase change material for thermal energy storage applications. Sol. Energy Mater. Sol. Cells.

[4] Qi G.-Q., Liang C.-L., Bao R.-Y., Liu Z.-Y., Yang W., Xie B.-H., Yang M.-B. (2014). Polyethylene glycol based shape-stabilized phase change material for thermal energy storage with ultra-low content of graphene oxide. Sol. Energy Mater. Sol. Cells.

[5] Qi G.-Q., Yang J., Bao R.-Y., Liu Z.-Y., Yang W., Xie B.-H., Yang M.-B. (2015). Enhanced comprehensive performance of polyethylene glycol based phase change material with hybrid graphene nanomaterials for thermal energy storage. Carbon.

[6] Liu Z., Wei H., Tang B., Xu S., Shufen Z. (2018). Novel light–driven CF/PEG/SiO_2_ composite phase change materials with high thermal conductivity. Sol. Energy Mater. Sol. Cells.

[7] Sarı A., Biçer A., Alkan C. (2017). Thermal energy storage characteristics of poly(styrene-co-maleic anhydride)-graft-PEG as polymeric solid–solid phase change materials. Sol. Energy Mater. Sol. Cells.

[8] Guo C., Chen Y., Li L. (2018). Investigation on interfacial interaction and thermal properties of flame retarded wood-plastic form-stable phase change material. Compos. Interfaces.

[9] Marcovich N.E., Reboredo M.M., Aranguren M.I. (1998). Dependence of the mechanical properties of woodflour–polymer composites on the moisture content. J. Appl. Polym. Sci..

[10] Hu S., Chen W., Liu W., Li H. (2007). Microwave irradiation treatment of wood flour and its application in PVC-wood flour composites. J. Wuhan Univ. Technol. -Mater Sci. Ed..

[11] Liu R., Cao J., Peng Y., Chen Y. (2015). Physical, mechanical, and thermal properties of micronized organo-montmorillonite suspension modified wood flour/poly(lactic acid) composites. Polym. Compos..

[12] Ma L., Guo C., Ou R., Sun L., Wang Q., Li L. (2018). Preparation and Characterization of Modified Porous Wood Flour/Lauric-Myristic Acid Eutectic Mixture as a Form-Stable Phase Change Material. Energy Fuels.

[13] Jiang L., Lei Y., Liu Q., Wang Y., Zhao Y., Lei J. (2019). Facile preparation of polyethylene glycol/wood-flour composites as form-stable phase change materials for thermal energy storage. J. Therm. Anal. Calorim..

[14] Song J., Chen C., Zhu S., Zhu M., Dai J., Ray U., Li Y., Kuang Y., Li Y., Quispe N. (2018). Processing bulk natural wood into a high-performance structural material. Nature.

[15] Nguyen V.D., Nguyen T.T., Zhang A., Hao J., Wang W. (2019). Effect of three tree species on UV weathering of wood flour-HDPE composites. J. For. Res..

[16] Zhao P., Guo C., Li L. (2018). Exploring the effect of melamine pyrophosphate and aluminum hypophosphite on flame retardant wood flour/polypropylene composites. Constr. Build. Mater..

[17] Kou Y., Wang S., Luo J., Sun K., Zhang J., Tan Z., Shi Q. (2019). Thermal analysis and heat capacity study of polyethylene glycol (PEG) phase change materials for thermal energy storage applications. J. Chem. Thermodyn..

[18] Liu C., Yao A., Chen K., Shi Y., Feng Y., Zhang P., Yang F., Liu M., Chen Z. (2021). MXene based core-shell flame retardant towards reducing fire hazards of thermoplastic polyurethane. Compos. Part B Eng..

[19] Liu C., Shi Y., Feng Y., You X., Xie W., Ke Y., Wang H., Chen L., Wang Y. (2022). Design of core-multi shell flame retardant towards fire safe thermoplastic polyurethane composites with low toxic fumes generation. Compos. Commun..

[20] Shi Y., Wang Z., Liu C., Wang H., Guo J., Fu L., Feng Y., Wang L., Yang F., Liu M. (2022). Engineering titanium carbide ultra-thin nanosheets for enhanced fire safety of intumescent flame retardant polylactic acid. Compos. Part B Eng..

[21] Wang W., Peng Y., Chen H., Gao Q., Li J., Zhang W. (2017). Surface microencapsulated ammonium polyphosphate with beta-cyclodextrin and its application in wood-flour/polypropylene composites. Polym. Compos..

[22] Umemura T., Arao Y., Nakamura S., Tomita Y., Tanaka T. (2014). Synergy Effects of Wood Flour and Fire Retardants in Flammability of Wood-plastic Composites. Energy Procedia.

[23] Utracki L.A., Sepehr M., Boccaleri E. (2007). Synthetic, layered nanoparticles for polymeric nanocomposites (PNCs). Polym. Adv. Technol..

[24] Liu R., Peng Y., Cao J. (2016). Thermal stability of organo-montmorillonite-modified wood flour/poly(lactic acid) composites. Polym. Compos..

[25] Barick A.K., Tripathy D.K. (2011). Effect of organically modified layered silicate nanoclay on the dynamic viscoelastic properties of thermoplastic polyurethane nanocomposites. Appl. Clay Sci..

[26] Giannelis E.P. (1996). Polymer layered silicate nanocomposites. Adv. Mater..

[27] Madhoushi M., Chavooshi A., Ashori A., Ansell M.P., Shakeri A. (2013). Properties of wood plastic composite panels made from waste sanding dusts and nanoclay. J. Compos. Mater..

[28] Liu R., Cao J., Luo S., Wang X. (2013). Effects of two types of clay on physical and mechanical properties of poly(lactic acid)/wood flour composites at various wood flour contents. J. Appl. Polym. Sci..

[29] Albozahid M., Naji H.Z., Alobad Z.K., Saiani A. (2021). Effect of OMMT reinforcement on morphology and rheology properties of polyurethane copolymer nanocomposites. J. Elastomers Plast..

[30] Huang G., Gao J., Wang X. (2012). Preparation and characterization of montmorillonite modified by phosphorus–nitrogen containing quaternary ammonium salts. Appl. Surf. Sci..

[31] Wang Z., Wang C., Qin Y., Huang A., Liu R. (2020). Organo-montmorillonite modified wood flour/poly (lactic acid) composites via different modification process. Polym. Compos..

[32] Jang B.N., Wilkie C.A. (2005). The thermal degradation of polystyrene nanocomposite. Polymer.

[33] Jang B.N., Wilkie C.A. (2005). The effect of clay on the thermal degradation of polyamide 6 in polyamide 6/clay nanocomposites. Polymer.

[34] Costache M.C., Jiang D.D., Wilkie C.A. (2005). Thermal degradation of ethylene–vinyl acetate coplymer nanocomposites. Polymer.

[35] Bahreyni E., Farid M., Fakhari M.A., Farid M. (2017). Ammonium Polyphosphate and Organically-Modified Montmorillonite Synergistic Effect on Flame-Retardant and Foaming Properties of High Density Polyethylene/Walnut Shell Powder Biocomposite. Iran. J. Polym. Sci. Technol..

[36] Zheng J., Ozisik R., Siegel R.W. (2006). Phase separation and mechanical responses of polyurethane nanocomposites. Polymer.

[37] Song Y., Wang N., Li J., Shan X., Zou G., Zhao C. (2019). Synergistic effect of organic montmorillonite and lignin-based charring agent with phosphorus on flame retardant poly(lactic acid). Fuhe Cailiao Xuebao/Acta Mater. Compos. Sin..

[38] Shi Y., Liu C., Duan Z., Yu B., Liu M., Song P. (2020). Interface engineering of MXene towards super-tough and strong polymer nanocomposites with high ductility and excellent fire safety. Chem. Eng. J..

[39] Liu C., Wu W., Shi Y., Yang F., Liu M., Chen Z., Yu B., Feng Y. (2020). Creating MXene/reduced graphene oxide hybrid towards highly fire safe thermoplastic polyurethane nanocomposites. Compos. Part B Eng..

[40] Liu C., Xu K., Shi Y., Wang J., Ma S., Feng Y., Lv Y., Yang F., Liu M., Song P. (2022). Fire-safe, mechanically strong and tough thermoplastic Polyurethane/MXene nanocomposites with exceptional smoke suppression. Mater. Today Phys..

[41] Liu C., Yang D., Sun M., Deng G., Jing B., Wang K., Shi Y., Fu L., Feng Y., Lv Y. (2022). Phosphorous-Nitrogen flame retardants engineering MXene towards highly fire safe thermoplastic polyurethane. Compos. Commun..

[42] Kamarian S., Yu R., Song J.-i. (2021). Synergistic effects of halloysite nanotubes with metal and phosphorus additives on the optimal design of eco-friendly sandwich panels with maximum flame resistance and minimum weight. Nanotechnol. Rev..

[43] Tirri T., Aubert M., Wilén C.-E., Pfaendner R., Hoppe H. (2012). Novel tetrapotassium azo diphosphonate (INAZO) as flame retardant for polyurethane adhesives. Polym. Degrad. Stab..

[44] Mandlekar N., Cayla A., Rault F., Giraud S., Salaun F., Guan J. (2020). Development of Novel Polyamide 11 Multifilaments and Fabric Structures Based on Industrial Lignin and Zinc Phosphinate as Flame Retardants. Molecules.

[45] Schartel B., Wilkie C.A., Camino G. (2016). Recommendations on the scientific approach to polymer flame retardancy: Part 2—Concepts. J. Fire Sci..

[46] Shi Y., Liu C., Fu L., Feng Y., Lv Y., Wang Z., Liu M., Chen Z. (2021). Highly efficient MXene/Nano-Cu smoke suppressant towards reducing fire hazards of thermoplastic polyurethane. Compos. Part A: Appl. Sci. Manuf..

[47] Zhang S., Ji W., Han Y., Gu X., Li H., Sun J. (2018). Flame-retardant expandable polystyrene foams coated with ethanediol-modified melamine-formaldehyde resin and microencapsulated ammonium polyphosphate. J. Appl. Polym. Sci..

[48] Lu X., Liang B., Sheng X., Yuan T., Qu J. (2020). Enhanced thermal conductivity of polyurethane/wood powder composite phase change materials via incorporating low loading of graphene oxide nanosheets for solar thermal energy storage. Sol. Energy Mater. Sol. Cells.

[49] Wang C., Feng L., Li W., Zheng J., Tian W., Li X. (2012). Shape-stabilized phase change materials based on polyethylene glycol/porous carbon composite: The influence of the pore structure of the carbon materials. Sol. Energy Mater. Sol. Cells.

[50] Zhang D., Tian S., Xiao D. (2007). Experimental study on the phase change behavior of phase change material confined in pores. Sol. Energy.

[51] Liang B., Lu X., Li R., Tu W., Yang Z., Yuan T. (2019). Solvent-free preparation of bio-based polyethylene glycol/wood flour composites as novel shape-stabilized phase change materials for solar thermal energy storage. Sol. Energy Mater. Sol. Cells.

[52] Shi Y., Liu C., Liu L., Fu L., Yu B., Lv Y., Yang F., Song P. (2019). Strengthening, toughing and thermally stable ultra-thin MXene nanosheets/polypropylene nanocomposites via nanoconfinement. Chem. Eng. J..

[53] Shi Y., Gui Z., Yuan B., Hu Y., Zheng Y. (2017). Flammability of polystyrene/aluminim phosphinate composites containing modified ammonium polyphosphate. J. Therm. Anal. Calorim..

[54] Qin P., Yi D., Xing J., Zhou M., Hao J. (2021). Study on flame retardancy of ammonium polyphosphate/montmorillonite nanocompound coated cellulose paper and its application as surface flame retarded treatment for polypropylene. J. Therm. Anal. Calorim..

[55] Yi D., Yang R. (2010). Ammonium polyphosphate/montmorillonite nanocompounds in polypropylene. J. Appl. Polym. Sci..

[56] Chen Y., Li M., Hao F., Yang C. (2022). Enhanced flame retardant performance of rigid polyurethane foam by using the modified OMMT layers with large surface area and ammonium polyphosphate. Mater. Today Commun..

[57] Liu C., Shi Y., Ye H., He J., Lin Y., Li Z., Lu J., Tang Y., Wang Y., Chen L. (2023). Functionalizing MXene with Hypophosphite for Highly Fire Safe Thermoplastic Polyurethane composites. Compos. Part A Appl. Sci. Manuf..

[58] Bee S.-T., Lim K.-S., Sin L.T., Ratnam C.T., Bee S.L., Rahmat A.R. (2018). Interactive effect of ammonium polyphosphate and montmorillonite on enhancing flame retardancy of polycarbonate/acrylonitrile butadiene styrene composites. Iran. Polym. J..

[59] Liu C., Zhang P., Shi Y., Rao X., Cai S., Fu L., Feng Y., Wang L., Zheng X., Yang W. (2020). Enhanced Fire Safety of Rigid Polyurethane Foam via Synergistic Effect of Phosphorus/Nitrogen Compounds and Expandable Graphite. Molecules.

